# Electroacupuncture modulates electroencephalographic microstate dynamics to alleviate chronic insomnia: a machine learning approach for predicting individual treatment response

**DOI:** 10.3389/fneur.2026.1782826

**Published:** 2026-02-27

**Authors:** Enqi Liu, Chi Wang, Xiaoqiu Wang, Kai Liu, Shan Qin, Liyu Lin, Juan Li, Min Xu, Chengyong Liu, Huangan Wu, Wenzhong Wu

**Affiliations:** 1 Jiangsu Province Hospital of Chinese Medicine, Affiliated Hospital of Nanjing University of Chinese Medicine, Nanjing, Jiangsu, China; 2 Yueyang Hospital of Integrated Traditional Chinese and Western Medicine, Shanghai University of Traditional Chinese Medicine, Shanghai, China; 3 Shanghai Institute of Acupuncture and Meridian, Shanghai, China; 4 Sleep and Psychology Center, Nanjing Municipal Hospital of Chinese Medicine, Nanjing University of Chinese Medicine, Nanjing, Jiangsu, China

**Keywords:** chronic insomnia, EEG microstates, electroacupuncture, machine learning, treatment-outcome prediction model

## Abstract

**Background:**

Chronic insomnia (CI) is associated with dysregulation of brain network dynamics, and patient response to electroacupuncture (EA) treatment varies. This study aimed to investigate the characteristics of electroencephalographic (EEG) microstates in patients with CI, analyze changes in microstate parameters before and after EA treatment, and explore the potential application of machine learning (ML) models based on baseline microstate features for predicting treatment response.

**Methods:**

We enrolled 41 CI patients and 19 healthy controls (HC). Baseline resting-state EEG was recorded, and microstate parameters (classes A–D) were analyzed. CI patients underwent 4-week EA treatment. Six clinical scales—including the Pittsburgh Sleep Quality Index (PSQI) and Hamilton Depression Scale, and microstate dynamics were compared pre- and post-treatment. Treatment response was defined as ≥50% PSQI reduction. Multi-stage feature selection and eight ML algorithms were used to build the prediction model.

**Results:**

At baseline, CI patients showed differences in some temporal metrics of microstates B, A, and C compared to HC. After EA, all clinical scores improved significantly (*p* < 0.001). Coverage_B and Duration_B, as well as Occurrence_C, increased, and multiple transition probabilities were regulated—particularly, microstate B temporal indicators normalized to HC levels. In the exploratory ML modeling, RF performed best (AUC = 0.849). “Duration_A,” “OrgTM_D → B,” and “OrgTM_C → B” were the top positive predictors, while “Occurrence_C” and “Duration_B” were negative predictors.

**Conclusion:**

This study found that EA treatment was associated with improved clinical scores and alterations in some EEG microstate parameters in patients with CI. In this exploratory analysis with a limited sample size, baseline microstate features showed preliminary potential for predicting treatment response, though further validation in larger cohorts is needed. These findings may provide a reference for future research on neurophysiological predictors and the development of individualized treatment strategies.

## 1 Introduction

Chronic insomnia (CI) is a prevalent sleep disorder characterized by persistent difficulties in initiating or maintaining sleep, accompanied by daytime impairments. Epidemiological studies indicate that CI affects approximately 10–15% of the global adult population, representing a significant public health burden (1, 2). The condition of CI patients can significantly affect their cognitive function, emotional regulation, and overall quality of life. Additionally, the severity of insomnia correlates with an increased risk of developing comorbid psychiatric disorders and cardiovascular diseases (3, 4). In clinical practice, symptoms of CI are commonly alleviated by pharmacological agents such as benzodiazepines and Z-drugs (5), or managed through cognitive behavioral therapy for insomnia (CBT-I) (6). However, the complex and heterogeneous etiology of CI, combined with the potential for side effects, dependency, or limited accessibility of these standard treatments, often undermines long-term therapeutic efficacy and patient adherence (7, 8). Therefore, it is imperative to explore alternative and complementary interventions to more effectively manage this disorder.

Various viable alternatives have been investigated for the treatment of CI, among which electroacupuncture (EA) has been increasingly adopted for its holistic regulatory approach and favorable safety profile (9–11). In China, EA is recommended as a complementary therapy for sleep disorders, including insomnia (12). Neuroimaging and electrophysiological studies have demonstrated that EA can modulate dysregulated neural circuits and oscillatory activity associated with sleep–wake cycles, thereby facilitating improvements in sleep architecture and subjective sleep quality (13, 14).

To elucidate the electrophysiological basis of this modulation, techniques such as EEG have become important research tools. Recent research has employed periodic and aperiodic EEG features to assess the modulatory effects of acupuncture on brain dynamics. For instance, parameterizing EEG power spectra into periodic components (e.g., adjusted alpha power) and an aperiodic component (i.e., the aperiodic exponent) allows for a finer-grained analysis of acupuncture-induced neural changes. This approach provides a more comprehensive evaluation beyond traditional band-power analysis by separately quantifying oscillatory activity and background 1/f dynamics (15). Based on functional network analysis utilizing phase synchronization metrics such as the phase lag index, acupuncture has been shown to significantly enhance whole-brain synchronization within specific frequency bands—particularly in the *δ* and *α* bands—and to optimize the topological architecture of functional networks. This is specifically manifested in the strengthening of their small-world network properties, thereby improving the efficiency of information integration and transmission (16). Furthermore, advanced methods like low-dimensional neural manifold decoding have been used to characterize brain state transitions under acupuncture stimulation, exploring this within the framework of an “acupuncture-brain interface.” (17) For example, some studies have attempted to use deep learning models (e.g., Transformer networks) to directly identify specific acupuncture manipulation states from scalp EEG (18). A functional magnetic resonance imaging meta-analysis focusing on mild cognitive impairment has also provided spatial evidence supporting acupuncture’s modulatory effects on key brain regions and networks (19). Collectively, this multi-faceted evidence suggests that acupuncture possesses the potential to reshape brain network dynamics.

It is also noteworthy that individual differences in resting-state brain network dynamics are associated with clinical symptoms and treatment responses in sleep disorders, as shown by previous neurophysiological examinations (20, 21). Recent research indicates that EEG microstates—brief, recurrent patterns of global neural synchronization—can reflect these large-scale network dynamics and may distinguish between different clinical profiles (22, 23). EEG microstates are characterized as semi-stable, topographical maps of scalp voltage that persist for tens of milliseconds before rapidly transitioning, representing the coordinated activity of distributed neural assemblies (24–27). On the relationship between EEG microstates and the treatment of neuropsychiatric conditions, studies have shown that microstate parameters can change following therapeutic interventions (28–30). Additionally, other research has indicated that baseline microstate features may predict response to treatments such as antidepressants (31, 32). This implies that EEG microstates hold promise as potential biomarkers for assessing and predicting the effectiveness of therapeutic interventions.

Existing studies have preliminarily identified abnormalities in EEG microstates among patients with CI. Compared to healthy controls (HC), individuals with insomnia exhibit shorter durations of microstate B, lower occurrence and coverage of microstates B and C, as well as higher occurrence and coverage of microstates D and E (33). However, another study reported reduced occurrence and contribution of microstate D (34). These findings remain largely descriptive and inconsistently replicated across studies, with the underlying relationship between such microstate alterations and response to clinical interventions yet to be elucidated. Furthermore, although multiple randomized controlled trials have demonstrated the efficacy of acupuncture in improving insomnia, its specific mechanisms of modulating brain network dynamics remain poorly understood. Crucially, there is a lack of objective neurophysiological markers capable of predicting individual treatment responses prior to intervention.

In this context, this study proposes an integrative research framework combining EEG microstate analysis with machine learning (ML) modeling to preliminarily explore the association between EA and changes in brain microstate dynamics in patients with CI and to attempt to construct an exploratory predictive model based on baseline EEG features. We hypothesize that CI patients exhibit characteristic microstate abnormalities, that EA can modulate these aberrant dynamics, and that baseline microstate parameters can effectively predict clinical response. By comparing resting-state microstate features between patients and HC, analyzing changes in microstate parameters before and after treatment, and employing multi-stage feature selection and ML algorithms for exploratory modeling, this study aims to provide preliminary data for understanding the relationship between EA intervention and brain dynamics, and to offer a reference for future exploration of neural predictors for individualized therapy.

## 2 Materials and methods

### 2.1 Subjects

This study included 41 patients with CI and 19 age-matched HC, all recruited from the Department of Acupuncture and Rehabilitation at Jiangsu Province Hospital of Chinese Medicine. The inclusion criteria for CI patients were: (1) aged 18–60 years; (2) meeting the diagnostic criteria for CI disorder according to the International Classification of Sleep Disorders, Third Edition (ICSD-3) (35); (3) Pittsburgh Sleep Quality Index (PSQI) score between 5 and 16 (36); (4) no current use of psychotropic medications; (5) absence of communication or cognitive impairments; and (6) provision of written informed consent. Exclusion criteria comprised: (1) severe neuroendocrine, cardiovascular, cerebrovascular, hematopoietic, or oncological diseases; (2) sleep disorders secondary to major depression, anxiety disorders, schizophrenia, or other serious mental illnesses, with a Hamilton Anxiety Scale (HAMA) score > 14 and/or Hamilton Depression Scale (HAMD) score > 17 (37, 38); (3) other sleep disorders such as obstructive sleep apnea, Rapid Eye Movement sleep behavior disorder, or restless legs syndrome; (4) contraindications to acupuncture treatment, including pregnancy or lactation; (5) receipt of acupuncture treatment for insomnia within the past month; (6) history of alcohol and/or substance abuse or dependence; and (7) inability to cooperate with EEG monitoring or presence of contraindications to EEG. All participants were right-handed and provided written informed consent. The study protocol was approved by the Medical Ethics Committee of the Affiliated Hospital of Nanjing University of Chinese Medicine (Approval No.: 2022 N-205-02; Approval Date: January 4, 2023) and conducted in accordance with the Declaration of Helsinki.

### 2.2 Study design and assessments

Resting-state EEG data were acquired from all participants. Clinicians administered the PSQI to both CI patients and HC. Additionally, CI patients were evaluated using the HAMA, HAMD, Insomnia Severity Index (ISI), Fatigue Severity Scale (FSS), and Hyperarousal Scale (HAS) to assess emotional disturbances and daytime functioning. Following a 4-week intervention comprising 12 EA sessions, post-treatment resting-state EEG data and clinical scale assessments were collected again from the CI patients.

### 2.3 EA intervention

**Figure 1:**
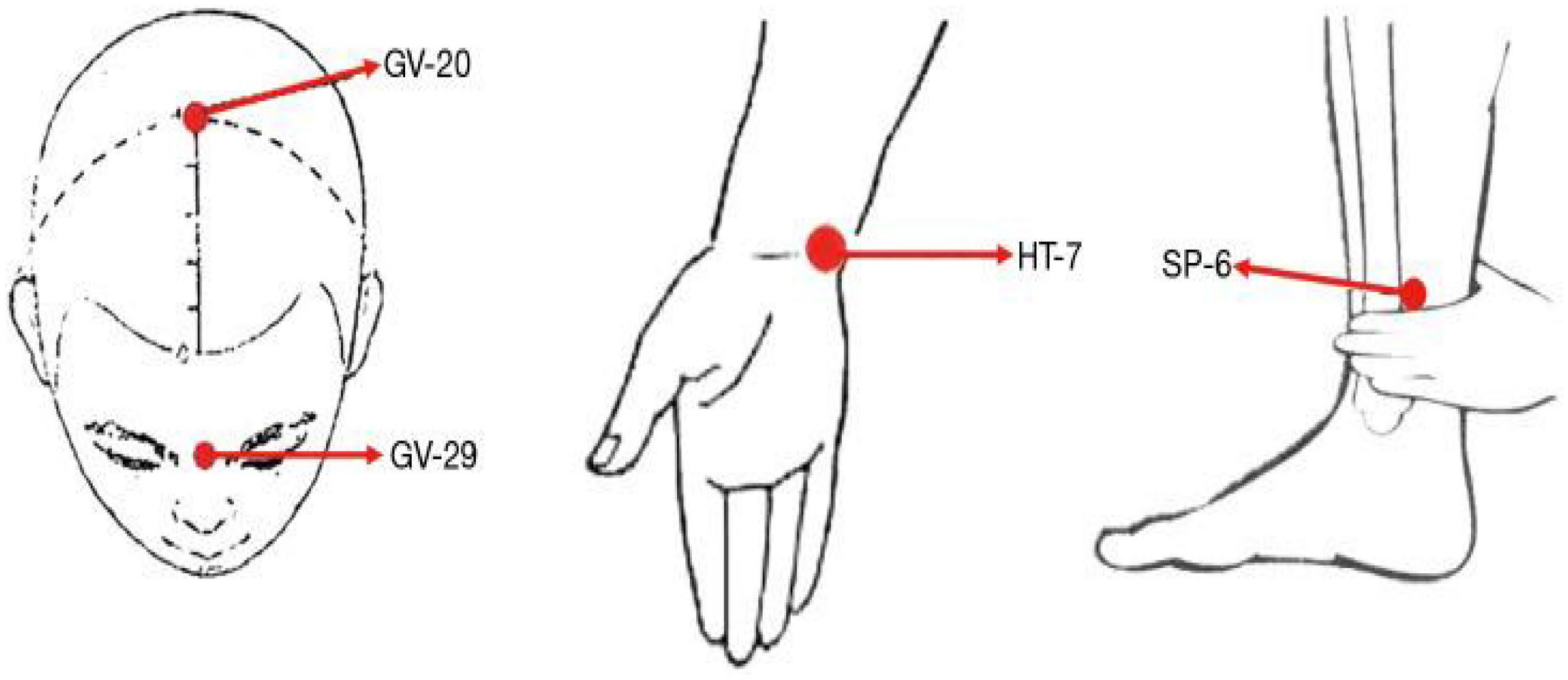
Schematic diagram of the locations of acupuncture acupoints.

All acupuncture procedures were conducted by licensed acupuncturists. The acupoints selected included Baihui (GV20), Yintang (EX-HN3), bilateral Shenmen (HT7), and bilateral Sanyinjiao (SP6; Figure 1). CI participants were placed in a supine position. After routine disinfection, disposable sterile acupuncture needles were inserted to appropriate depths based on the patient’s body size and acupoint location. Manual manipulation was applied to elicit *deqi*, a sensation characterized by soreness, numbness, or distension around the needle site. Subsequently, the needles at Baihui and Yintang were connected to an EA device (Huatuo SDZ-IIB, Suzhou Medical Appliance Factory, China). A continuous wave stimulation at 2 Hz was delivered at an intensity tolerable to the patient. The stimulation lasted for 30 min, after which all needles were removed. The treatment was administered every other day, three times per week, for a total of 4 weeks, resulting in 12 sessions.

### 2.4 EEG data acquisition

Resting-state EEG signals were acquired using a 26-channel wireless EEG system (Natus Medical, America; Supplementary Figure S1). Electrodes were positioned according to the international 10–20 system. The amplifier bandwidth was set to 1–70 Hz, and the sampling rate was 512 Hz. Electrode impedance was maintained below 10 kΩ, with the reference electrode placed at the vertex (Cz). All recordings were conducted in a quiet and comfortable room. Participants wore earplugs to minimize interference from ambient temperature and noise.

Prior to EA treatment, resting-state EEG was recorded under both eyes-open and eyes-closed conditions. Each condition lasted 1 min, and participants were instructed to remain relaxed and avoid head or body movement. The two conditions were alternated three times, yielding a total recording duration of 6 min. The same EEG acquisition procedure was repeated for CI patients after completing the 12 treatment sessions.

### 2.5 EEG data preprocessing

All EEG data were preprocessed using the EEGLAB toolbox. To reduce interference from visual input on endogenous resting-state brain activity, highlight intrinsic brain rhythms, and ensure data stability, only data recorded during the eyes-closed condition were used in this analysis (39, 40). The raw data were downsampled to 256 Hz, followed by band-pass filtering between 1 and 45 Hz and notch filtering between 48 and 52 Hz to remove power line interference. Subsequently, the continuous EEG data were segmented into 2-s non-overlapping epochs. After manual inspection, epochs containing artifacts were removed, and the remaining clean epochs were concatenated to reconstruct continuous EEG datasets. Channels with excessive noise were manually identified as bad channels and interpolated using data from adjacent channels. Independent Component Analysis (ICA) was employed to remove noise and artifact components (41). Specifically, ICA was used to identify and remove components associated with ocular movements, electrocardiographic, and electromyographic artifacts. On average, 5.58 ± 2.388 artifact components were removed for the CI group before treatment, 6.37 ± 2.283 after treatment, and 5.59 ± 2.280 for the HC group. No significant differences were found among the three groups (*p*_HC VS CI-PRE_ = 0.696, *p*_CI-PRE VSCI-POST_ = 0.717, *p*_HC VS CI-POST_ = 0.919). The criteria for component rejection were based on the component’s power spectrum, temporal characteristics, and visual comparison of its topography with canonical artifact templates. To improve computational efficiency and for microstate analysis, a secondary band-pass filter was applied within the 2 to 20 Hz range (34), and the EEG was re-referenced to the average reference.

After preprocessing, the CI group before treatment retained an average of 148.29 ± 20.876 s of artifact-free EEG data, the CI group after treatment 150.10 ± 22.041 s, and the HC group 150.74 ± 26.876 s. No significant differences were observed between the groups (*p*_HC VS. CI-PRE_ = 0.992, *p*_CI-PRE VS. CI-POST_ = 0.123, *p*_HC VS. CI-POST_ = 0.215).

### 2.6 EEG microstate analysis

Microstate analysis was conducted on EEG signals under three conditions: HC at baseline, CI patients before treatment, and CI patients after treatment. All analyses were performed using EEGLAB (v2024.0) in combination with the MicrostateLAB plugin (42).

First, the global field power (GFP) was calculated from 26-channel resting-state EEG data. Instantaneous topographic maps at GFP peaks were extracted and used as inputs for subsequent clustering to improve signal-to-noise ratio and topographic stability (43). Second, an unsupervised K-means clustering algorithm (with 100 random restarts) was applied to cluster these GFP peak maps into 4 classes; maps with inverted polarity were treated as the same class (44). This process was repeated for each participant to obtain individual-level microstate topographic sequences. Subsequently, all individual maps were subjected to another K-means clustering across participants (ignoring polarity) to generate group-level average microstate templates. Finally, the group-level templates were back-fitted to GFP peaks from each participant: each peak was assigned to a microstate class based on the maximum absolute spatial correlation. This procedure yielded a time-state sequence for each subject, from which the following microstate metrics were derived: Coverage_X, Duration_X, Occurrence_X, and Organizational Transition Matrix (OrgTM)_X → Y.

### 2.7 ML based predictive modeling

#### 2.7.1 Feature selection

To construct a predictive model for the efficacy of EA in treating CI, a multistage feature selection strategy was adopted to identify the most predictive EEG microstate indicators. Patients with CI who showed a reduction of >50% in PSQI scores after EA treatment were defined as responders, forming the basis for the classification outcome (34).

Three ML methods were applied for feature screening: Random Forest (RF) (using Gini impurity for feature importance) (45), Recursive Feature Elimination (RFE) with logistic regression to iteratively remove redundant features (46), and Extreme Gradient Boosting (XGBoost) to evaluate feature contributions (47). Each method independently selected the top 15 features. Stability of feature selection was assessed via bootstrap resampling (100 iterations), retaining features selected in >75% of resampled sets. The intersection of features identified by at least two methods was determined using a Venn diagram. Multicollinearity was addressed by excluding features with variance inflation factor (VIF) > 10 or Pearson correlation coefficient >0.8. The resulting feature subset exhibited high robustness and low redundancy. All analyses were conducted in Python 3.13 using scikit-learn (48), XGBoost, and statsmodels.

#### 2.7.2 Model construction and interpretability analysis

Multiple ML algorithms were employed to develop the predictive model, including CatBoost, XGBoost (47), Logistic Regression (LR), Support Vector Machine (SVM) (49), RF (45), k-Nearest Neighbors (KNN), Naive Bayes (NB), and AdaBoost. To address limited sample size and class imbalance, bootstrap oversampling of the minority class was performed until balance with the majority class was achieved. This bootstrap oversampling strategy was employed to address the class imbalance caused by the small sample size in this exploratory study, with the full recognition that it may potentially overestimate the model’s performance in subsequent evaluation and limit the direct extrapolation of the results. RF and XGBoost were configured with 100 trees (n_estimators), CatBoost with 100 iterations, and k-Nearest Neighbors (KNN) with 5 neighbors (n_neighbors). To ensure reproducibility, all models were run with random_state = 42. Where applicable, n_jobs = −1 was set to enable parallel computing for improved efficiency, while other hyperparameters retained their default values.

Model evaluation employed stratified random splitting (Stratified Shuffle Split) with 100 repetitions, maintaining consistent responder/non-responder ratios in training (75%) and testing (25%) sets. Performance was assessed using area under the receiver operating characteristic curve (AUC), accuracy, precision, recall, and F1-score. ROC curves with confidence intervals were generated, and DeLong’s test (50) was used to evaluate statistical significance of AUC differences.

To explain the optimal model (the RF with the highest cross-validation AUC), SHapley Additive exPlanations (SHAP) were applied to visualize feature contributions and dependencies. All analyses were performed using Python 3.13.

### 2.8 Statistical analysis

Demographic characteristics were compared between groups (HC and CI). For quantitative data, the Shapiro–Wilk test was used to assess the normality of continuous variables. If the data conformed to a normal distribution, they were expressed as mean ± standard deviation (SD); if not, they were expressed as median and interquartile range (IQR). For categorical data, they were expressed as rates or proportions. Gender distribution was analyzed using the chi-square test. Age, years of education, and PSQI scores were compared between groups using independent-samples *t*-tests. Within-group comparisons of clinical scales—including PSQI, ISI, FSS, and HAS—before and after treatment in CI patients were performed using paired *t*-tests.

Following microstate analysis, topographic maps for four microstate classes (A, B, C, and D) were derived across conditions. For each template, four microstate parameters were computed: duration, coverage, occurrence, and transition probability. A two-way repeated-measures analysis of variance (rmANOVA) was conducted for duration, coverage, and occurrence, with group and microstate class as factors (51). In cases of significant main or interaction effects, *post-hoc* paired comparisons were performed using Dunn’s test. Data are presented as mean ± SD. The statistical significance of 12 transition probabilities was assessed using paired *t*-tests.

To control the family-wise error rate, the Bonferroni correction was applied, with adjusted *p*-values < 0.05 considered statistically significant. All statistical analyses were conducted using SPSS version 27.0. Statistical significance was set at *p* < 0.05 or adjusted *p* < 0.05 when applicable.

## 3 Results

### 3.1 Demographic characteristics and clinical scale scores

Demographic characteristics of both the HC and CI groups are summarized in Table 1. No significant differences were observed between the two groups in age, gender, years of education (all *p* > 0.05).

**Table 1:** Demographic data of CI patients and HC.

Characteristic	HC(*n* = 19)	CI(*n* = 41)	*t/z/x^2^*	*p*
Age	34.9 ± 8.346	34.56 ± 11.711	−1.991	0.51
Years of education	16.8 ± 2.440	16.37 ± 2.31	1.043	0.301
PSQI	4(2)	13.00(3)	−4.895	<0.001
Sex (Male/Female)	8/11	18/23	0.17	0.896

Compared to baseline, CI patients showed significant improvements after treatment in PSQI, ISI, FSS, HAMA, HAMD, and HAS scores (all *p* < 0.001). The results are presented graphically in Table 2.

**Table 2:** Clinical scale score changes of CI patients and HC.

Clinical scale	CI-Pre-EA(*n* = 41)	CI-Post-EA(*n* = 41)	*t/z*	*p*
PSQI	13.00(3)	7(5)	13.249	<0.001
ISI	16.93 ± 4.74	11.073 ± 4.51	9.070	<0.001
HAS	40.71 ± 9.03	36.29 ± 8.53	7.160	<0.001
FSS	47.05 ± 9.63	40.12 ± 9.40	7.399	<0.001
HAMD	10.20 ± 3.00	6.95 ± 2.75	8.123	<0.001
HAMA	9.68 ± 2.54	6.85 ± 2.59	5.993	<0.001

### 3.2 Abnormalities in EEG microstate spatial topography and temporal parameters in CI patients

**Figure 2:**
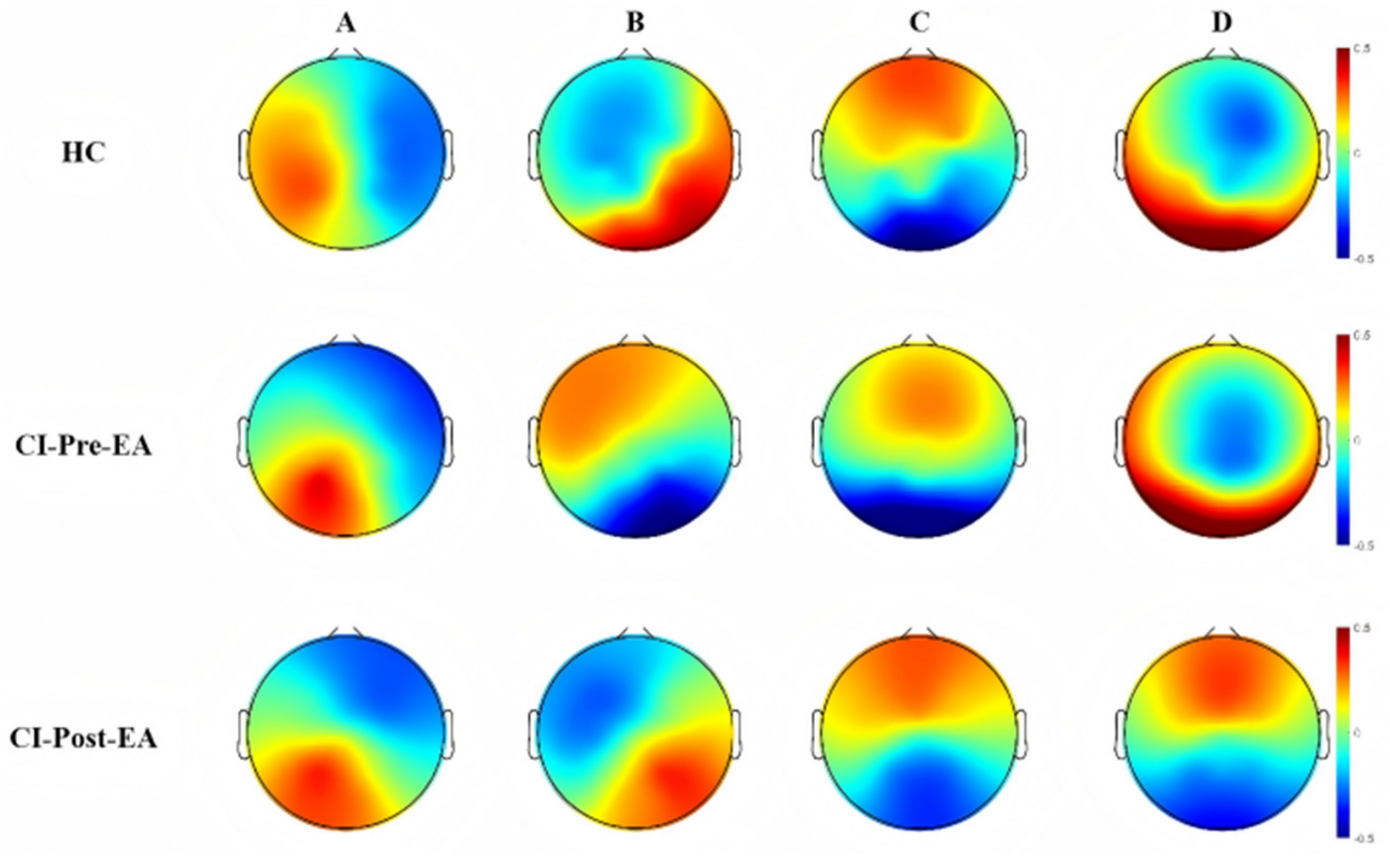
Microstate clustering results of the CI group before and after treatment and the HC group. Among them: CI-pre-EA (*n* = 41) denotes the CI group before receiving EA treatment, CI-post-EA (*n* = 41) denotes the CI group after receiving EA treatment, and HC (*n* = 19) denotes the healthy control group.

Figure 2 presents the group-level microstate topographic maps for CI patients before treatment, CI patients after treatment, and HC. The clustering analysis identified four canonical microstate classes—labeled A, B, C, and D—independent of polarity. The spatial configurations of these microstates were consistent with those reported in previous studies (23). Specifically, microstate A exhibited a left occipital to right frontal orientation, microstate B displayed a right occipital to left frontal orientation, microstate C showed a relatively symmetric fronto-occipital orientation, and microstate D was characterized by a central frontal maximum. Topographic analysis of variance performed on the microstate maps across the three groups revealed no significant differences in any microstate class between conditions (all *p* > 0.05).

**Figure 3:**
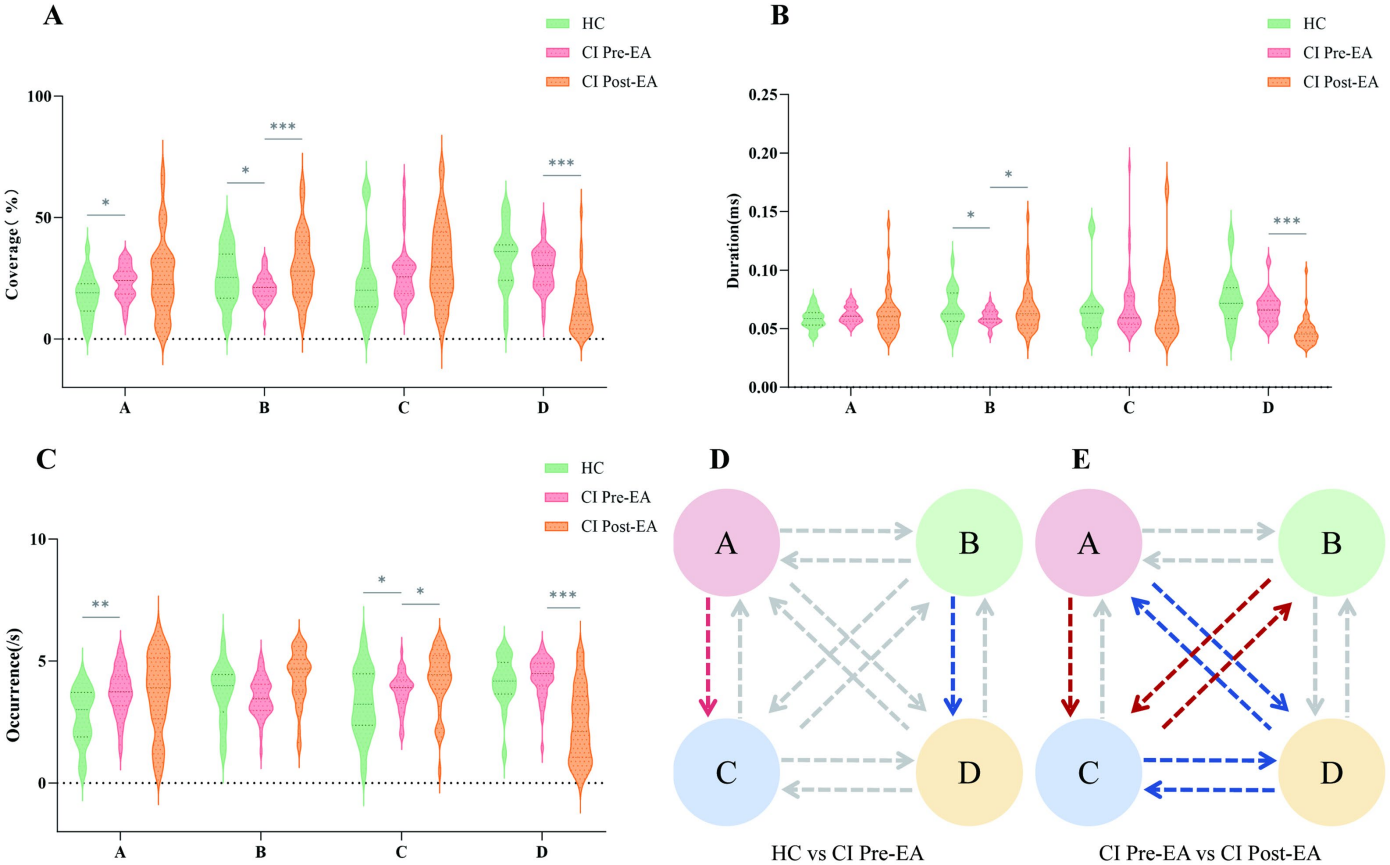
Differences in microstate characteristic parameters between the CI group before and after treatment and the healthy control group: **(A)** Coverage, **(B)** duration, **(C)** occurrence. **(D)** OrgTM between CI before treatment and HC; **(E)** OrgTM in CI before vs. after treatment. CI Pre-EA (*n* = 41) denotes the CI group before receiving EA treatment, CI Post-EA (*n* = 41) denotes the CI group after receiving EA treatment, and HC (*n* = 19) denotes the healthy control group. The significance markers were the results after Bonferroni correction. *p < 0.05, **p < 0.01, ***p < 0.001. Red curves indicate higher TP values in the latter group; blue curves indicate lower TP values in the latter group.

As shown in Figures 3A–C and Table 3, the coverage and occurrence of microstate A, as well as the Occurrence_C, were significantly higher in patients with CI than in HC (*p* < 0.05), whereas the coverage and duration of microstate B were significantly lower (*p* < 0.05). Furthermore, compared with the HC, the OrgTM_A → C and OrgTM_B → D in the CI group were also significantly higher (*p* < 0.05; Figure 3D; Table 4).

**Table 3:** Microstate parameters (Mean ± SD) of CI group before and after treatment and HC group.

Microstate parameter	HC (*n* = 19)	Pre-EA (*n* = 41)	Post-EA (*n* = 41)	*p*	
				HC vs. Pre-EA	Pre-EA vs. Post-EA
Coverage_A	17.66 ± 8.82	23.09 ± 6.87	20.86 ± 14.19	0.012*	0.437
Coverage_B	21.37 ± 6.87	21.12 ± 6.87	27.83 ± 16.99	0.031*	<0.001***
Coverage_C	23.42 ± 16.16	26.83 ± 11.48	34.73 ± 18.41	0.353	0.131
Coverage_D	33.00 ± 12.88	28.95 ± 8.52	16.59 ± 12.32	0.153	<0.001***
Duration_A	58.38 ± 8.64	62.01 ± 7.25	57.67 ± 14.96	0.096	0.777
Duration_B	66.85 ± 16.53	59.75 ± 6.82	66.08 ± 29.04	0.021*	0.039*
Duration_C	66.52 ± 26.49	69.04 ± 26.39	76.36 ± 34.97	0.733	0.746
Duration_D	75.97 ± 22.73	67.19 ± 12.99	51.14 ± 18.45	0.062	<0.001***
Occurrence_A	2.89 ± 1.21	3.69 ± 0.97	3.28 ± 1.61	0.008**	0.159
Occurrence_B	3.71 ± 1.12	3.51 ± 0.80	3.93 ± 1.33	0.436	0.088
Occurrence_C	3.25 ± 1.34	3.84 ± 0.74	4.38 ± 1.37	0.030*	0.030*
Occurrence_D	4.24 ± 1.05	4.28 ± 0.88	2.89 ± 1.50	0.883	<0.001***

The significance markers were the results after Bonferroni correction. *p < 0.05, **p < 0.01, ***p < 0.001.

**Table 4:** Original transition probabilities of CI group before and after treatment and HC group.

Original transition probabilities	HC (*n* = 19)	Pre-EA (*n* = 41)	Post-EA (*n* = 41)	*p*	
				HC vs. Pre-EA	Pre-EA vs. Post-EA
OrgTM_A → B	6.13 ± 3.19	6.52 ± 2.45	6.79 ± 2.45	1.000	1.000
OrgTM_A → C	5.57 ± 3.20	8.01 ± 2.66	9.69 ± 1.04	0.036*	*p* < 0.001***
OrgTM_A → D	8.19 ± 4.30	9.29 ± 3.63	5.56 ± 5.81	1.000	*p* < 0.001 ***
OrgTM_B → A	6.06 ± 3.08	6.65 ± 2.40	6.94 ± 4.27	1.000	1.000
OrgTM_B → C	7.88 ± 5.77	7.56 ± 2.81	13.82 ± 1.43	1.000	*p* < 0.001***
OrgTM_B → D	12.65 ± 7.95	8.60 ± 2.38	6.76 ± 5.92	0.047*	0.152
OrgTM_C → A	5.71 ± 3.23	7.98 ± 2.72	9.56 ± 6.73	0.076	0.668
OrgTM_C → B	7.82 ± 5.62	7.58 ± 2.87	13.93 ± 9.30	1.000	*p* < 0.001 ***
OrgTM_C → D	9.58 ± 5.45	10.02 ± 3.47	7.33 ± 5.06	1.000	*p* < 0.01**
OrgTM_D → A	8.08 ± 4.28	9.16 ± 3.48	5.52 ± 5.85	1.000	*p* < 0.001 ***
OrgTM_D → B	12.59 ± 7.79	8.67 ± 2.66	6.80 ± 6.12	0.063	0.181948
OrgTM_D → C	9.74 ± 5.39	9.97 ± 3.35	7.31 ± 5.26	1.000	*p* < 0.01**

The significance markers were the results after Bonferroni correction. *p < 0.05, **p < 0.01, ***p < 0.001.

### 3.3 EA modulated abnormal microstate parameters in CI patients

The microstate parameters of the CI group before and after treatment are presented in Tables 3, 4 and Figures 3A–C,E. After 4 weeks of 2-Hz EA treatment, the coverage and duration of microstate B, as well as the occurrence rate of microstate C, in CI significantly increased (*p* < 0.05); meanwhile, the coverage, duration, and occurrence rate of microstate D decreased. Additionally, the transition probabilities between microstate B and C (B↔C) and OrgTM_A → C increased, whereas the transition probabilities between microstate A and D (A↔D) and between microstate C and D (C↔D) decreased.

### 3.4 Prediction of EA treatment outcomes based on baseline EEG microstates

**Figure 4:**
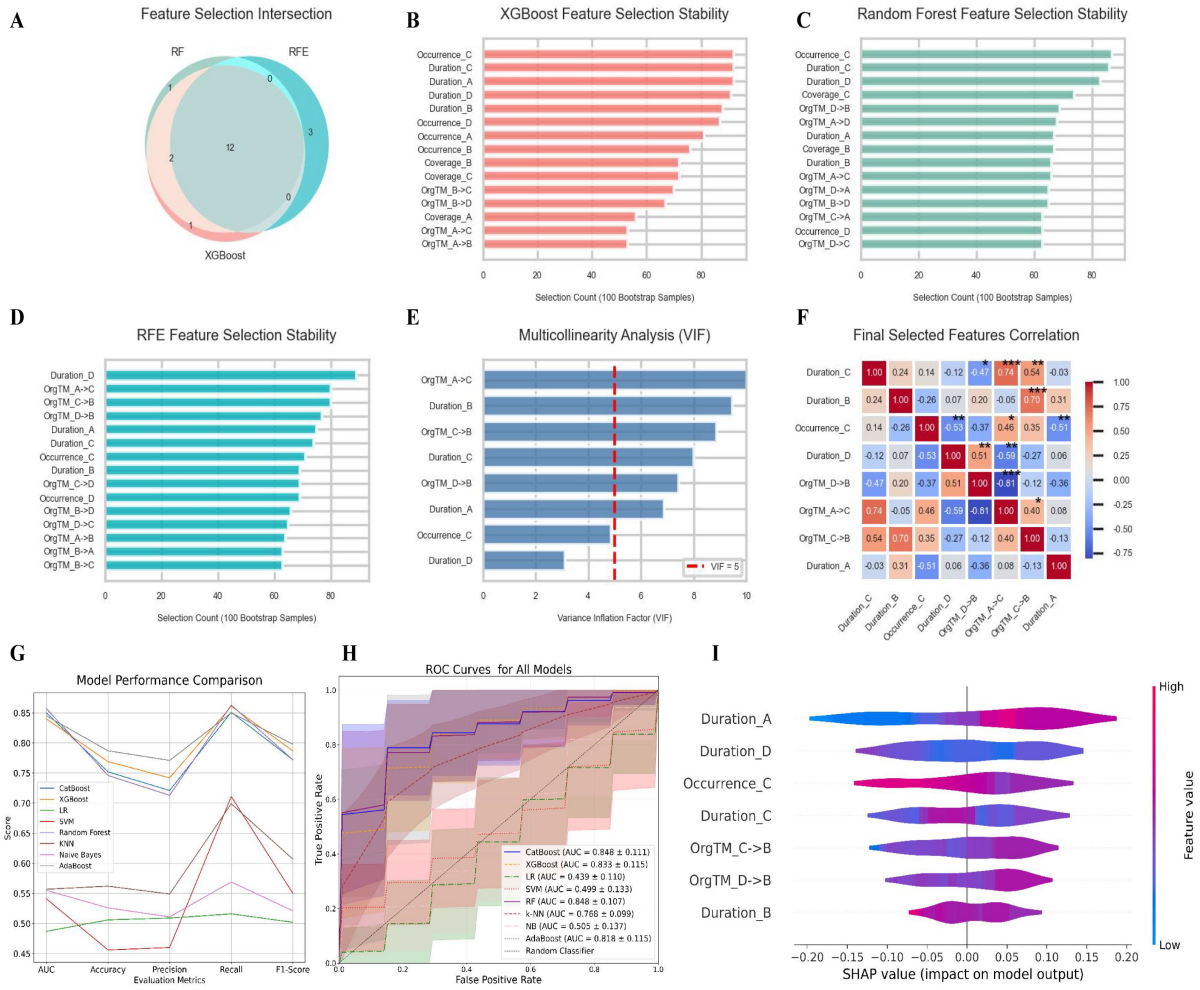
Multistage feature selection, model performance comparison, and SHAP-based interpretability analysis for predicting EA response in CI: **(A)** Feature selection intersection among three methods. Venn diagram illustrating the overlap of top 15 features selected by RF, RFE, and XGBoost methods. The central intersection represents 12 features commonly identified by all three algorithms, forming the initial candidate feature set for downstream modeling. **(B)** Stability of feature selection by XGBoost across bootstrap resampling. Bar plot showing the frequency with which each feature was selected across 100 bootstrap resampling iterations using the XGBoost algorithm. Higher bar lengths indicate greater selection stability, reflecting consistent importance across different subsamples. **(C)** Stability of feature selection by RF across bootstrap resampling. Bar plot depicting the number of times each feature was selected in 100 bootstrap resampling runs using the RF algorithm. Features with higher counts are more consistently selected, indicating robustness in their predictive relevance. **(D)** Stability of feature selection by RFE across bootstrap resampling. Bar plot representing the selection frequency of each feature across 100 bootstrap resampling iterations using the RFE method. Features appearing frequently across resamples demonstrate high reproducibility and reliability in feature ranking. **(E)** Multicollinearity analysis using VIF. Bar plot showing the VIF values for each candidate feature. A red dashed line marks the threshold of VIF = 5; features exceeding this value are considered to exhibit significant multicollinearity and are candidates for removal. **(F)** Correlation matrix of final selected features. Heatmap displaying Pearson correlation coefficients among the final selected features after removing highly correlated or multicollinear variables. Colors range from blue (negative correlation) to red (positive correlation), with asterisks indicating statistically significant correlations (*p* < 0.05). **(G)** Comparison of model performance across evaluation metrics. Line plot comparing the average performance of eight ML models across five evaluation metrics: AUC, accuracy, precision, recall, and F1 score. Each point represents the mean value over 100 stratified cross-validation runs, with error bars omitted for clarity. **(H)** ROC curves for all models. Stacked ROC curves for eight ML models, with shaded areas representing 95% confidence intervals derived from 100 cross-validation repetitions. The AUC and its SD are reported for each model. **(I)** SHAP values for key features in the RF model. Violin plots showing the distribution of SHAP values for six key features in the optimal RF model. Positive SHAP values indicate increased prediction probability of treatment response, while negative values suggest decreased likelihood. The width of the violin reflects the density of SHAP values at each level.

To preliminarily explore the possibility of predicting the efficacy of EA in treating CI, we conducted exploratory ML modeling based on the available data. Patients who showed a reduction of >50% in PSQI scores after EA treatment were defined as responders, serving as the classification label. Among the 41 patients with CI, 14 were defined as responders and 27 as non-responders (34.1, 65.9%). To address class imbalance, an oversampling technique was applied to balance both classes to 27 cases each (50.50%). A multi-stage feature selection strategy was employed to identify a robust and non-redundant set of predictive features from initial EEG microstate metrics. As illustrated in Figure 4A, 12 features commonly selected by RF, RFE, and XGBoost formed a preliminary feature subset. Among these, “Duration_B,” “Duration_D,” “Duration_C,” “Occurrence_C,” “OrgTM_D → B,” “OrgTM_C → B,” and “Duration_A” demonstrated high stability across bootstrap iterations (Figures 4B–D). The feature “OrgTM_C → B” was excluded due to a VIF exceeding 10 and a correlation coefficient with “OrgTM_D → B” greater than 0.8 (Figures 4E,F). Subsequent multicollinearity and correlation analyses confirmed that the remaining features all had VIF values below the threshold of 10, indicating no severe multicollinearity, and most pairwise Pearson correlation coefficients were below 0.7, supporting feature stability and model interpretability.

Using the refined feature set, we constructed and evaluated eight ML models. In this exploratory small-sample analysis, the RF classifier outperformed others across all evaluation metrics, achieving a mean AUC of 0.849 ± 0.133 (the performance may be overestimated due to the use of oversampling; Figure 4G). Other well-performing models included XGBoost (AUC = 0.833 ± 0.115) and CatBoost (AUC = 0.848 ± 0.111), whereas traditional algorithms such as logistic regression and naïve Bayes showed inferior performance across multiple metrics (Figure 4H). Without the use of oversampling, the models performed poorly on the original dataset (AUC = 0.461 ± 0.144, Precision-Recall Curve Under the Area Curve (PR-AUC) = 0.431 ± 0.097), with the latter being nearly equivalent to the random-guessing baseline (Precision = 0.341), confirming that class imbalance substantially impaired the model’s discriminative ability. After oversampling, the AUC improved to 0.849 ± 0.133, and the PR-AUC increased significantly to 0.876 ± 0.088 (substantially exceeding the balanced baseline of 0.500; see Supplementary Figure S2). To interpret the optimal model’s decision-making process, we performed SHAP analysis on the RF model. The resulting violin plot (Figure 4I) visualized the contribution of each feature to the output, identifying “Duration_A,” “OrgTM_D → B,” and “OrgTM_C → B” as the most important positive predictors, while “Occurrence_C” and “Duration_B” exhibited negative influences. Given the small sample size, overfitting risk and potential performance overestimation due to oversampling, these findings tentatively suggest that specific EEG microstate parameters may be associated with treatment response and could serve as preliminary neurophysiological candidate biomarkers for large-scale, multi-center future exploration.

## 4 Discussion

By leveraging a combination of EEG microstate analysis and ML, the present study aimed to address two core objectives: to investigate the neurophysiological changes associated with EA treatment for CI before and after intervention, and to preliminarily develop a predictive model for identifying individualized treatment responses. Our key findings revealed three pivotal observations: First, patients with CI exhibited markedly abnormal baseline temporal dynamics in microstates, characterized by significantly higher coverage and occurrence of microstate A, as well as a higher occurrence of microstate C, along with significantly lower coverage and duration of microstate B. Additionally, dynamic abnormalities in microstate transitions were observed in these patients, which may be associated with dysregulated resting-state brain networks in this population. Second, during the four-week EA intervention, the coverage and duration of microstate B showed a trend toward normalization to the levels observed in HC, while corresponding adjustments were also noted in the temporal characteristics of microstates C and D. Third, baseline microstate features, particularly microstate durations and specific inter-microstate transition probabilities, demonstrated predictive potential for treatment response as measured by PSQI score improvement in this study. Collectively, these findings offer new observational perspectives on the neurophysiological changes accompanying EA treatment for CI, while also suggesting that microstate parameters may have potential biomarker value for predicting individualized treatment responses.

This study employed the classic A–D four-class microstate model for analysis. This choice was based on dual considerations of methodological maturity and alignment with the research objectives. First, this model represents the most classic and widely adopted analytical framework in resting-state EEG microstate research. According to existing literature, the four canonical microstate classes are thought to potentially map onto the activity of large-scale brain networks; for instance, microstates A, B, C, and D are often associated with the auditory network, visual network, SN, and attention network, respectively (29, 52, 53). Research indicates (34, 54, 55) that the four-class model can effectively capture the core dynamic patterns of large-scale brain networks, providing a foundation for its applicability in this study (56). Second, to develop a ML model that is robust, interpretable, and holds potential for clinical prediction, utilizing the well-validated four-class features as inputs helps ensure the comparability of analytical results and provides a clear neuroscientific basis for interpreting the model. In the present study, we observed multi-dimensional abnormalities in the temporal parameters of microstates in patients with CI. These aberrant patterns may collectively constitute the characteristic disturbances in resting-state brain network dynamics associated with CI. Specifically, CI patients exhibited increased coverage and occurrence of microstate A. Given that microstate A is frequently associated with the ventral attention network (VAN) in the literature (57)—a network whose functions include external environmental perception and attentional orienting—we speculate that this abnormality may reflect a “hyperarousal” neural signature specific to CI. This may suggest that, even in the eyes-closed resting state, the brains of CI patients may remain in a state of heightened vigilance, unconsciously enhancing the processing of internal and external sensory signals (58, 59).

Meanwhile, compared with healthy participants, CI patients showed reduced coverage and duration of microstate B, alongside hyperactivation of microstate C as evidenced by its increased occurrence. This pattern aligns with previous research (33, 60): following sleep deprivation, microstate B was suppressed and microstate C exhibited hyperactivation, with the occurrence rate of microstate B showing a significant negative correlation with Subjective sleepiness (e.g., Karolinska Sleepiness Scale scores). Furthermore, the increased occurrence rate of microstate C in patients with CI may be related to its overactivation, which is implicated in processing personally salient information and self-referential thinking (26). This elevation in microstate C occurrence is also associated with functional abnormalities in the DMN and the executive control network (ECN)—a finding supported by the work of Khoo S. Y. et al. (61). It is noteworthy that reduced duration of microstate B has been linked to prolonged reaction times in psychomotor vigilance tasks, increased subjective/objective sleepiness, and decreased positive affect, suggesting that it may serve as a potential neurodynamic indicator linking nocturnal sleep disturbances to impaired daytime cognitive functions, such as attention. Prior studies have indicated that microstate C exhibits a competitive relationship with other microstate classes, suggesting it plays a unique role in the dynamics of canonical microstate transitions (33, 62). Additionally, we found that patients with CI showed a significant increase in transition probability from microstate A to C and a significant decrease in transition probability from microstate B to D, which may indicate a dysregulation in the dynamic coordination of brain networks in this population (34, 63).

Enhanced A → C transitions may reflect abnormal coupling between the SN and DMN, potentially leading to a tendency toward abnormal switching in CI patients between internal introspective states (e.g., DMN dominance) and states of readiness to respond to external stimuli (e.g., involving the SN and central executive network) (64). In contrast, reduced B → D transitions may suggest decreased efficiency in the brain’s dynamic shift from sensory processing to cognitive control. Collectively, these microstate anomalies may represent one potential neurophysiological substrate underlying clinical manifestations of CI, such as impaired cognitive flexibility and attentional regulation deficits (65).

Our study found that after 4 weeks of EA, scores on the PSQI, ISI, FSS, HAMA, HAMD, and HAS were significantly improved—this is consistent with our previously reported significant efficacy of EA in treating CI (9, 66, 67). However, the mechanism underlying EA’s therapeutic effect in CI requires further investigation. Our team previously reported that after EA treatment, CI patients exhibited reduced high-frequency EEG activity, increased low-frequency EEG activity, and decreased entropy values, suggesting that modulating EEG signals in CI patients may be one of the mechanisms by which EA improves sleep (9). Nevertheless, whether and how EA modulates EEG microstates—a key indicator reflecting the dynamic coordination of large-scale brain networks—remains unclear. The present study provides new electrophysiological observations for this: after 4 weeks of EA treatment, the originally abnormal parameters of microstate B (coverage and duration) in CI patients showed a trend toward normalization, approaching levels observed in HC. Microstate B is primarily associated with the DMN and visual network, and typically dominates during rest, introspection, and relaxation. The occurrence rate of microstate C, which is often associated with the SN and attentional network activity, was altered following EA treatment. Building on this association, the changes in microstate B parameters suggest that EA intervention may facilitate a shift in brain activity toward a state more inclined to internal focus and relaxation, thereby potentially creating conditions conducive to sleep initiation. The acupuncture point prescription employed in this study (Baihui GV20, Yintang EX-HN3, bilateral Shenmen HT7, and Sanyinjiao SP6) was selected based on traditional Chinese medicine (TCM) theory and modern neuroanatomical evidence. This multi-site combination has been widely used in previous studies for insomnia treatment, and its effects may be associated with the modulation of multiple neurophysiological processes involved in sleep–wake regulation (68). Based on TCMtheory, Baihui (GV20) and Yintang (EX-HN3) are located on the Governor Vessel and extraordinary points, respectively, and are commonly used for calming the spirit, stabilizing the mind, and regulating mental activity. Neuroimaging studies suggest that stimulation of these frontal scalp points can modulate activity in the prefrontal cortex and limbic system (69). Shenmen (HT7), located on the Heart Meridian, is a key point for nourishing the heart and calming the spirit. Studies have shown that stimulating this point can influence autonomic balance and emotional processing (70). Sanyinjiao (SP6) is the confluence point of the Liver, Spleen, and Kidney Meridians. It is used to nourish Yin and subdue Yang, aiming to correct the underlying imbalance considered in TCM theory to be responsible for insomnia (71). This combination of acupoints is designed to synergistically regulate the spirit, mental activity, and systemic balance, which corresponds to the multi-network modulation observed in the brain. Animal studies have demonstrated that EA at GV20, HT7, and SP6 may modulate the activity of the hypothalamic–pituitary–adrenal axis, affect various monoamine neurotransmitters (such as 5-HT), and influence neurotrophin pathways. Furthermore, a recent randomized controlled neuroimaging study also found that acupuncture can modulate abnormal functional connectivity between the hypothalamus—a core structure of the sleep–wake cycle—and the orbitofrontal cortex in patients with CI, and this change in connectivity is correlated with clinical improvement (72). One study found that acupuncture may improve clinical symptoms in patients with chronic insomnia by modulating the expression of specific genes related to neuroinflammation, thereby remodeling the functional connectivity of key brain regions, such as the striatum and prefrontal cortex (73). The treatment protocol utilized the even reinforcing-reducing needle technique combined with 2 Hz continuous-wave EA stimulation, which has frequently been reported in previous studies to be associated with sedative effects and the modulation of low-frequency neural oscillations (74–76). Thus, this multi-target stimulation protocol with specific parameters may be more inclined to exert multi-level modulation on the distributed brain network systems that maintain the balance between wakefulness and sleep (77, 78). This could thereby be associated with the clinical symptom improvements and microstate dynamic changes observed in the present study. Of course, this network modulation interpretation based on microstate changes remains a correlative finding and hypothesis; its definitive causal relationships and precise neural pathways require further in-depth validation in future studies incorporating control groups and more advanced multimodal techniques.

Although symptom alleviation was observed based on PSQI assessment following EA treatment, there remained significant inter-individual variability in therapeutic efficacy. Therefore, distinguishing potential responders before treatment is valuable for exploring individualized strategies. In recent years, studies have attempted to predict treatment responses in insomnia patients using baseline EEG features (51). For instance, Zhu Lin et al. (79) found that, among 36 patients with primary insomnia, the ML model based on baseline theta connectivity achieved the highest accuracy in predicting treatment response to repetitive transcranial magnetic stimulation (rTMS; AUC = 0.843). Another study by Yongjian Guo et al. (34) found that transition probabilities between microstates were useful for identifying patients with potential high responsiveness to repetitive rTMS treatment, with the final model achieving an average prediction accuracy of 80.13%. Following this line of reasoning, we exploratorily developed for the first time a predictive model for EA efficacy based on baseline microstate features. Among the models tested, the RF algorithm exhibited the optimal classification performance in this dataset (AUC = 0.849). Key predictive features included Duration_A, OrgTM_D → B, and OrgTM_C → B. These preliminary findings suggest that pre-treatment resting-state brain activity may contain information relevant to subsequent subjective treatment outcomes, potentially enabling the identification of patient subgroups most likely to benefit from EA. The ML methodology employed herein demonstrates distinct advantages over traditional statistical approaches in modeling high-dimensional, nonlinear relationships among microstate parameters, thereby enhancing predictive precision. The Precision-Recall curve is highly sensitive to class imbalance. If the performance improvement were merely due to overfitting, the PR-AUC should not have shown such a substantial leap. This result confirms that oversampling enabled the model to genuinely learn the characteristic patterns that distinguish responders from non-responders. SHAP analysis further improved model interpretability by quantifying feature contributions: Duration_A, OrgTM_D → B, and OrgTM_C → B emerged as critical positive predictors, whereas Occurrence_C and Duration_B served as negative predictors.

The preliminary results of this predictive framework may provide a preliminary theoretical reference for future exploration into pre-treatment prognostic assessment using neurophysiological indicators, yet its clinical translational potential is currently severely limited by the small sample size, the potential overestimation of model performance due to bootstrap oversampling, as well as the lack of external multi-center validation and the exclusive use of subjective PSQI scores (without objective polysomnography (PSG) sleep measurements) to define treatment response. For patients predicted to potentially respond poorly, future studies could explore the necessity of adjusting treatment plans—for instance, by adopting combined strategies such as integrating EA with pharmacotherapy or CBT-I, or implementing sequential multimodal interventions—in well-characterized cohorts with large sample sizes and objective sleep assessments.

While this study yielded several meaningful findings, it is not without limitations. First, this study adopted an open-label, single-arm, before-and-after comparison design without including a sham acupuncture or waitlist control group. Therefore, although significant improvements temporally associated with the EA intervention were observed, the potential influence of placebo effects, regression to the mean, or non-specific treatment factors cannot be fully excluded. Future studies require randomized, double-blind, placebo-controlled trial designs to confirm the specific efficacy of EA. Second, the machine learning predictive analysis in this study is strictly exploratory and preliminary in nature. The constructed prediction model is based on single-center data with a limited sample size, which directly leads to a significant risk of overfitting and severely restricts the model’s stability and generalization ability. Although bootstrap oversampling was employed to address class imbalance, this strategy may have potentially overestimated the model’s performance evaluation metrics (such as AUC and PR-AUC). Moreover, the model has not undergone any external multi-center validation, making its direct application to clinical practice impossible at this stage. Additionally, the definition of treatment responders relies entirely on subjective PSQI score reduction (≥50%) without incorporating objective polysomnographic sleep measures (such as sleep efficiency, total sleep time, and number of awakenings). This may introduce subjective bias into the classification outcomes and limit the reliability of the predictive model. This point is indirectly reflected in the performance variability of the model, as indicated by the large standard deviation of the reported AUC values (e.g., 0.849 ± 0.133 for the random forest model). Therefore, the clinical applicability of the current prediction model remains unclear. Future studies must utilize larger, multi-center datasets to validate the robustness of the model and consider incorporating objective PSG-derived metrics into the definition of treatment response to enhance its objectivity and reliability. Third, this study focused solely on resting-state EEG microstate features and did not incorporate EEG activity during sleep or acupuncture treatment sessions. Furthermore, the analysis primarily addressed linear microstate characteristics (e.g., duration, occurrence, and transition probabilities), overlooking their non-linear dynamic attributes. Subsequent research could integrate PSG recordings and EEG data collected during acupuncture sessions to comprehensively assess microstate dynamics across different sleep stages. Additionally, incorporating non-linear metrics of microstates (e.g., entropy rate, Lempel–Ziv complexity) could provide complementary insights. More importantly, the brain’s response to acupuncture is multifaceted. An analytical framework that extends beyond a single modality may reveal more complete mechanisms. Future studies could significantly enhance predictive accuracy and mechanistic understanding by constructing a multimodal EEG biomarker framework. For example, integrating the microstate temporal dynamics examined here with complementary spatio-temporal features—such as the periodic-aperiodic parameters of neural oscillations, measures of functional and effective brain network connectivity, and functional magnetic resonance imaging (fMRI) data—could provide a more comprehensive picture of brain activity, ranging from local rhythms and network synchronization to global dynamics. Integrating these multidimensional biomarkers within advanced ML pipelines holds great potential for developing robust, individualized neuromodulation strategies for CI. Finally, the definition of a “responder” as a participant with a ≥ 50% reduction in PSQI score, while clinically conventional, remains somewhat subjective. Future work may improve objectivity by incorporating PSG-derived measures—such as sleep efficiency and the number of awakenings—into the criteria for defining treatment response.

## 5 Conclusion

This study revealed EEG microstate dynamics associated with CI, characterized by significantly higher coverage and occurrence of microstate A, increased occurrence of microstate C, and notably lower coverage and duration of microstate B, along with dynamic abnormalities in microstate transitions. EA treatment was associated with changes in these abnormal patterns, with the parameters of microstate B showing a trend toward normalization. Based on these findings, an exploratory predictive model was constructed to identify responses to EA treatment for CI. This work provides preliminary clues for understanding brain dynamic responses under EA intervention and exploratorily explores the potential feasibility of using neurophysiological indicators for efficacy prediction, with no clinical application value at present. Future research is needed to validate these findings with more rigorous designs, larger multi-center sample sizes and objective sleep measurements, and to further investigate the underlying neural mechanisms.

## Data Availability

The original data supporting the conclusions of this article are available from the corresponding author upon obtaining permission from the corresponding author and will not be subject to unreasonable restrictions.

## Glossary

EEG: Electroencephalographic
CI: Chronic Insomnia
CBT-I: Cognitive behavioral therapy for insomnia
EA: Electroacupuncture
HC: Healthy Controls
ML: Machine Learning
DMN: Default Mode Network
SN: Salience Network
ICSD-3: International Classification of Sleep Disorders, Third Edition
PSQI: Pittsburgh Sleep Quality Index
HAMA: Hamilton Anxiety Scale
HAMD: Hamilton Depression Scale
ISI: Insomnia Severity Index
FSS: Fatigue Severity Scale
HAS: Hyperarousal Scale
ICA: Independent Component Analysis
GFP: Global Field Power
OrgTM: Organizational Transition Matrix
RF: Random Forest
RFE: Recursive Feature Elimination
XGBoost: Extreme Gradient Boosting
VIF: Variance Inflation Factor
LR: Logistic Regression
SVM: Support Vector Machine
AUC: Area Under the Receiver Operating Characteristic Curve
PR-AUC: Precision-Recall Curve Under the Area Curve
KNN: k-Nearest Neighbors
NB: Naive Bayes
SHAP: SHapley Additive exPlanations
SD: Standard Deviation
PSG: Polysomnography
IQR: Interquartile Range
rmANOVA: two-way repeated-measures analysis of variance
VAN: Ventral Attention Network
ECN: Executive Control Network
TCM: traditional Chinese medicine
rTMS: repetitive Transcranial Magnetic Stimulation
