# Supplementary material for: Electroacupuncture modulates electroencephalographic microstate dynamics to alleviate chronic insomnia: a machine learning approach for predicting individual treatment response
Source: Front Neurol. 2026 Feb 27;17:1782826. doi: 10.3389/fneur.2026.1782826 (PMC12982113; doi:10.3389/fneur.2026.1782826)
Supplement: Supplementary file 1 [file Data_Sheet_1.docx]

Supplementary Material


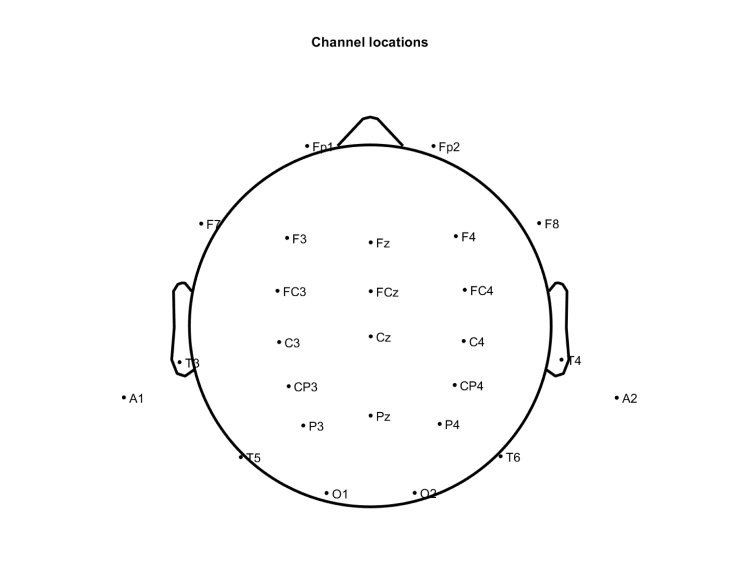


**Supplementary Figure S1.**26-lead electrode placement diagram.


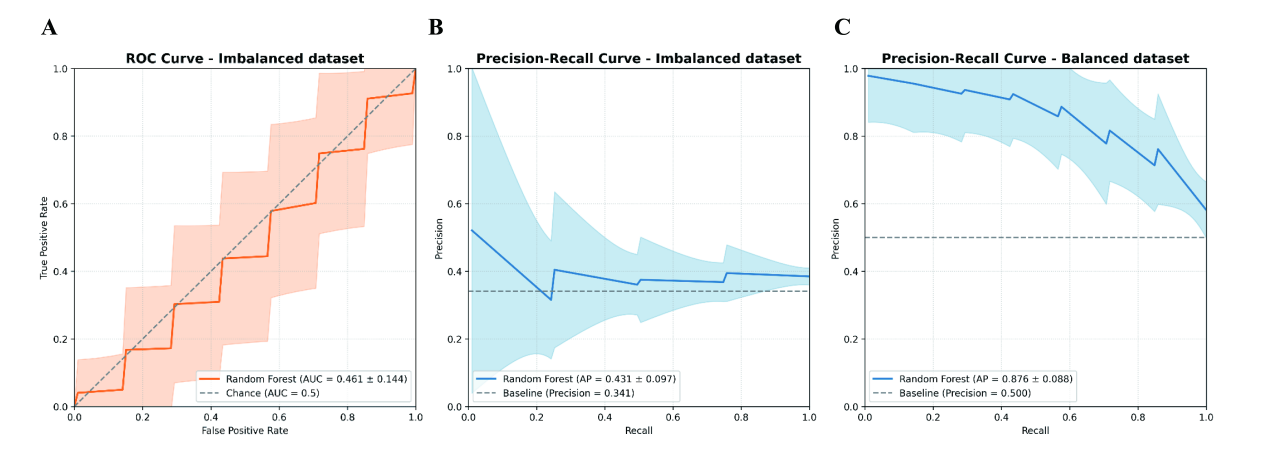


**Supplementary Figure S2.** Impact of class imbalance correction on Random Forest model performance. (A) ROC curve of the original imbalanced dataset. (B) Precision–Recall curve of the original imbalanced dataset. (C) Precision–Recall curve after balancing via oversampling.
